# Diffusion-weighted imaging for identifying patients at high risk of tumor recurrence following liver transplantation

**DOI:** 10.1186/s40644-019-0264-y

**Published:** 2019-11-15

**Authors:** Yi-Hsuan Chuang, Hsin-You Ou, Chun-Yen Yu, Chao-Long Chen, Ching-Chun Weng, Leo Leung-Chit Tsang, Hsien-Wen Hsu, Wei-Xiong Lim, Tung-Liang Huang, Yu-Fan Cheng

**Affiliations:** 1 Liver Transplantation Program, Department of Diagnostic Radiology, Kaohsiung Chang Gung Memorial Hospital, Chang Gung University College of Medicine, 123 Dapi Rd, Niaosong Dist, Kaohsiung, Taiwan, Republic of China; 2 Liver Transplantation Program, Department of Surgery, Kaohsiung Chang Gung Memorial Hospital, Chang Gung University College of Medicine, Kaohsiung, Taiwan

**Keywords:** Diffusion weighted imaging, ADC value, Tumor recurrence

## Abstract

**Background:**

Tumor recurrence is the major risk factor affecting post-transplant survival. In this retrospective study, we evaluate the prognostic values of magnetic resonance (MR) diffusion-weighted imaging (DWI) in liver transplantation for hepatocellular carcinoma (HCC).

**Methods:**

From April 2014 to September 2016, 106 HCC patients receiving living donor liver transplantation (LDLT) were enrolled. Nine patients were excluded due to postoperative death within 3 months and incomplete imaging data. The association between tumor recurrence, explant pathologic findings, and DWI parameters was analyzed (tumor-to-liver diffusion weighted imaging ratio, DWI_T/L_; apparent diffusion coefficients, ADC). The survival probability was calculated using the Kaplan–Meier method.

**Results:**

Sixteen of 97 patients (16%) developed tumor recurrence during the follow-up period (median of 40.9 months; range 5.2–56.5). In those with no viable tumor (*n* = 65) on pretransplant imaging, recurrence occurred only in 5 (7.6%) patients. Low minimum ADC values (*p* = 0.001), unfavorable tumor histopathology (*p* <  0.001) and the presence of microvascular invasion (*p* <  0.001) were risk factors for tumor recurrence, while ADC_mean_ (*p* = 0.111) and DWI_T/L_ (*p* = 0.093) showed no significant difference between the groups. An ADC_min_ ≤ 0.88 × 10^− 3^ mm^2^/s was an independent factor associated with worse three-year recurrence-free survival (94.4% vs. 23.8%) and overall survival rates (100% vs. 38.6%).

**Conclusions:**

Quantitative measurement of ADC_min_ is a promising prognostic indicator for predicting tumor recurrence after liver transplantation.

## Background

Liver transplantation (LT) is the preferred treatment for selected patients with hepatocellular carcinoma (HCC) and end-stage liver disease, given that it removes the tumor as well as the diseased liver. The Milan criteria [1], which considers the size and number of the tumors, was used for decades as the standard to determine transplant eligibility. Unfortunately, recurrence rates have been reported to be as high as 26% [2]. There is growing evidence that tumor biological behavior is a prognostic factor. Criteria applying various indicators such as alpha-fetoprotein (AFP), protein induced by vitamin K absence or antagonists-II (PIVKA-II), and the neutrophil–lymphocyte ratio (NLR) have been published [3–6]. Several studies have also confirmed that poor tumor differentiation and vascular invasion are correlated with early recurrence and worse outcomes [7, 8]. However, these tumor factors can only be obtained via explant pathology and thus may not be useful preoperatively.

Diffusion-weighted imaging (DWI) is a distinct functional imaging technique, allowing qualitative and quantitative assessment of the diffusion properties of various types of tissue. In highly cellular tumors, decreased extracellular space and increased tortuosity of the extracellular matrix hinder the mobility of water molecules. The apparent diffusion coefficient (ADC) values calculated from DWI data reflect the tumor microenvironment and have the potential to predict tumor histopathology.

The aim of this study was to investigate the association between pretransplant magnetic resonance (MR)-DWI indices and tumor factors with post-transplant recurrence.

## Methods

Institutional review board approval was obtained for this retrospective study of patient medical records; informed consent was waived.

### Study population

From April 2014 to September 2016, 240 patients consecutively underwent living donor liver transplantation (LDLT) at our institution. Of these, 106 had a confirmed diagnosis of HCC and five of them had separate intrahepatic cholangiocarcinoma (I-CC) or combined hepatocellular cholangiocarcinoma (cHCC-CC) in same liver after histological examination of the explant. Based on the MR imaging performed in 2 months prior to transplantation, all patients met the University of California San Francisco (UCSF) criteria [9]. We used AFP > 200 ng/mL as a cutoff [10, 11]. AFP elevation was a relative contraindication, and locoregional therapy, including radiofrequency ablation (RFA) and transarterial chemoembolization (TACE), were employed upon recognition of AFP elevation.

Pretransplant clinical parameters such as gender, age, underlying liver disease, model of end-stage liver disease (MELD) score, serum AFP level, and MR imaging data (number of tumors, tumor size, and DWI indices) were collected. The pathological findings analyzed were histologic differentiation and the presence of microvascular invasion. Five patients were excluded from the study due to postoperative death and four patients did not have DWI data. The median follow-up period was 40.9 months (range 5.2–56.5).

### MR imaging protocols

All MR images were acquired on a 1.5 T scanner (*Discovery MR450;* GE Healthcare, Milwaukee, WI, USA) and a twelve-channel body array coil for signal reception.

The MR protocols were the following: breath-hold in-phase and opposed-phase fast spoiled gradient-echo T1 weighted imaging; breath-hold fast spin-echo (FSE) T2-weighted imaging; single shot FSE heavily T2-weighted imaging with fat suppression; and diffusion-weighted imaging using SE single-shot echo-planar technique with b-values of 0 and 400 s/mm^2^ (repetition time/echo time (TR/TE): 2400/44; slice thickness/gap: 5/1 mm; matrix: 80 × 128). ADC maps were calculated automatically. Dynamic images using fat-suppressed T1-weighted gradient-echo images with a three-dimensional (3D) acquisition sequence (liver acquisition with volume acceleration [LAVA]) were obtained at 30 s, 60 s, and 3 min after IV administration of 0.2 mmol/kg gadopentetate dimeglumine. The images were acquired in the transverse plane and had a section thickness of 5 mm (TR/TE: 3.4/1.6; flip angle: 12°; matrix: 288 × 192).

### Imaging analysis

**Fig. 1:**
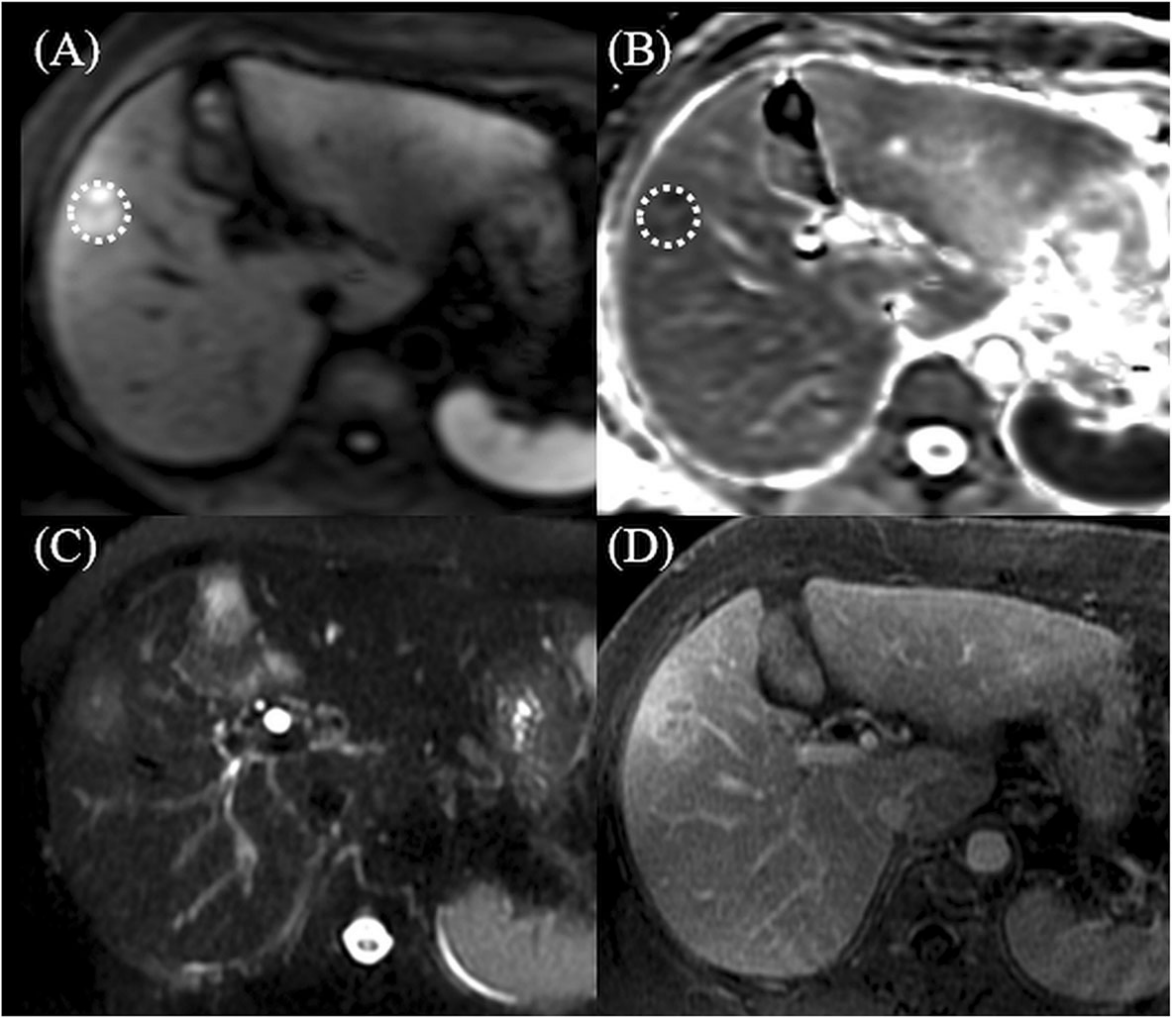
MR imaging in a 61-year-old woman. Pathologically confirmed grade 4 HCC. Dotted circles in **a** DWI and **b** ADC map show the placement of regions of interest. **c** T2-weighted imaging shows high signal intensity of the tumor. **d** Postcontrast T1-weighted image shows heterogeneous arterial enhancement with intralesional low SI area

**Fig. 2:**
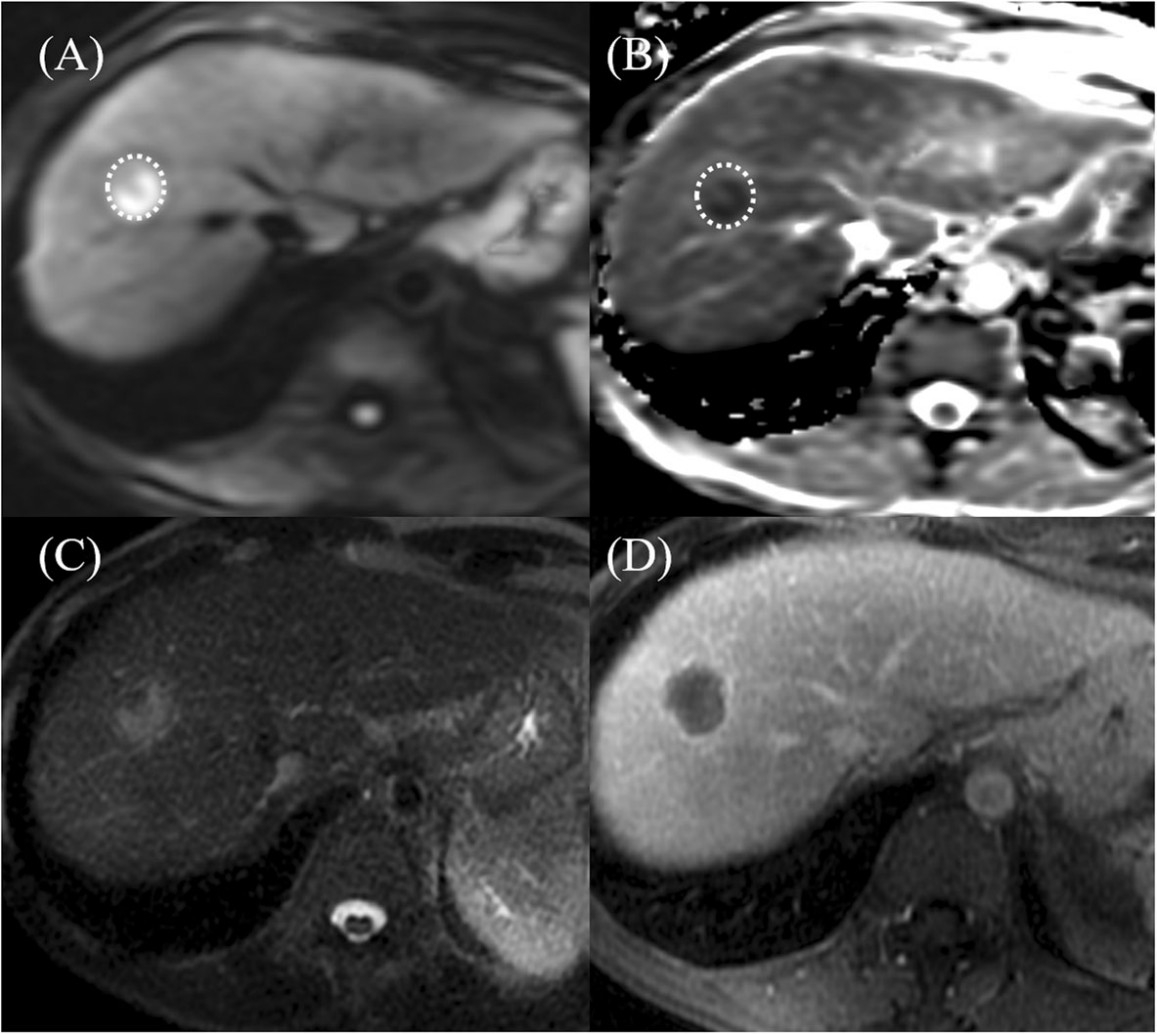
MR imaging in a 60-year-old man who underwent DEB-TACE procedure. Pathologically confirmed grade 4 HCC with 40% tumor necrosis. Dotted circles show the placement of regions of interest. **a** DWI and **b** ADC map show restricted diffusion area in the segment VIII tumor. **c** T2-weighted imaging. **d** Postcontrast T1-weighted image shows mildly enhancing viable tumor. ADC_min_ of tumor was 0.84 × 10^− 3^ mm^2^/s

**Fig. 3:**
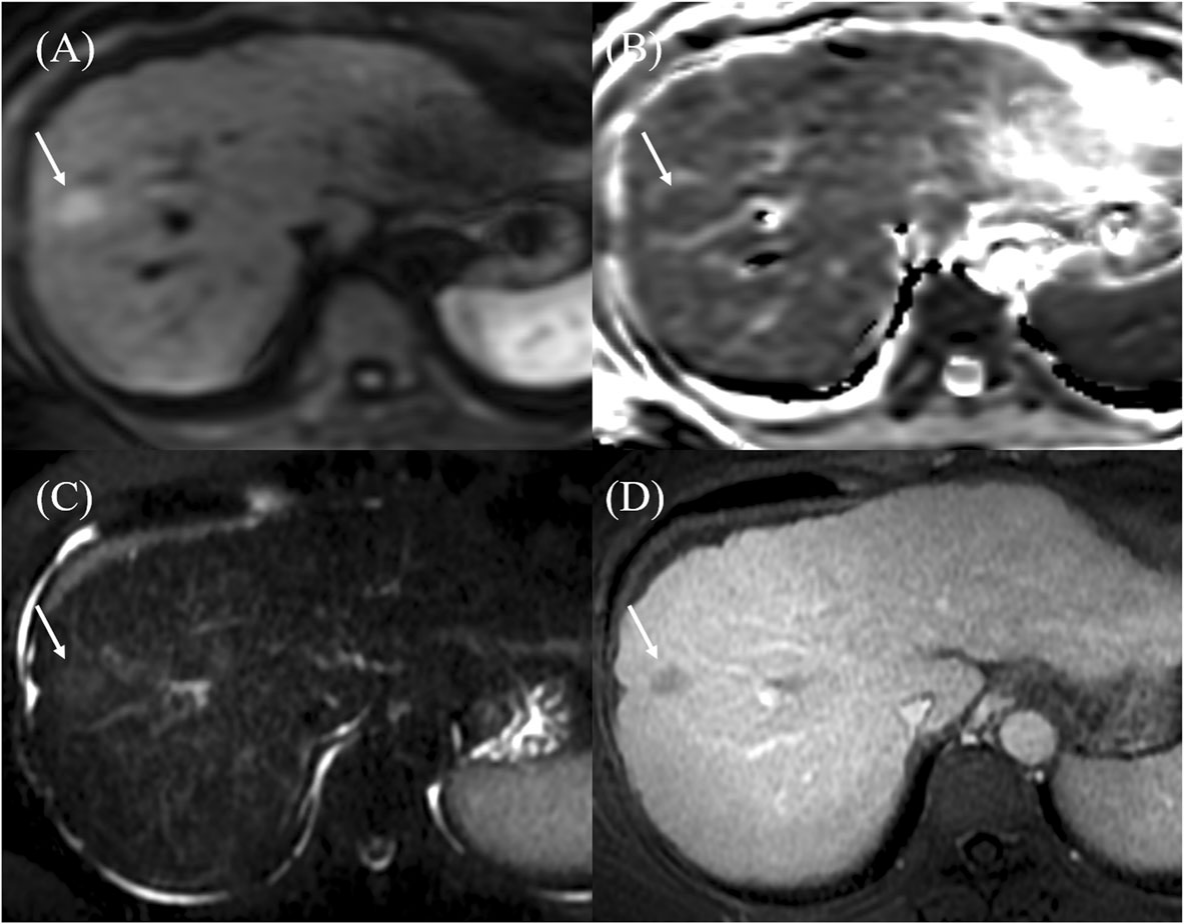
MR imaging in a 60-year-old woman. Pathologically confirmed grade 2 HCC. The tumor is hyperintense to surrounding liver parenchyma on **a** DWI (b = 400 s/mm^2^), slightly hypointense on **b** ADC map, and mild hyperintense on **c** T2-weighted imaging. **d** Postcontrast T1-weighted image shows minimal enhancement. ADC_min_ of tumor was 1.11 × 10^− 3^ mm^2^/s

MRI reports were interpreted by experienced radiologists (Cheng, Tsang, and Ou). Quantitative measurements were performed on the diffusion-weight imaging and ADC maps. The tumor-to-liver signal intensity (DWI_T/L_) ratio was calculated by dividing the signal intensity of the tumor by that of the liver. With respect to the mean and lowest ADC values of the tumor (ADC_mean_, ADC_min_), the regions of interest were manually placed at its maximum cross section of each viable tumor on a single slice, referencing T2-weighted and contrast-enhanced T1-weighted transverse images (Figs. 1, 2 and 3).

### Statistical analysis

Statistical analysis was performed using commercially available software (MedCalc, version 18.11, Ostend, Belgium). Clinicopathologic factors were compared between the nonrecurrence and recurrence groups. We used the Student’s *t*-test for continuous variables and the chi-square test for categorical variables. Cox proportional hazards regression was performed. Recurrence-free survival and overall survival rates were determined using the Kaplan–Meier method. The receiver operating characteristic curves for quantitative DWI indices were derived. All the *p*-values were two-sided, and *p* <  0.05 was considered statistically significant.

## Results

### Patient characteristics

The clinical data are summarized in Table 1. Sixty-five men and 32 women were included in this study. The mean age at transplant was 55.6 years. Eighty-eight (90%) patients had either hepatitis B virus (HBV) or hepatitis C virus (HCV) infection. Eighty-two patients (84%) had hepatectomy, RFA, or TACE before transplantation. Five patients met the UCSF criteria after the downstaging procedure. Tumor recurrence was observed in 16 cases (17%).

**Table 1:** Patient demographic and clinical characteristics

Variable	*N* = 97
Age (years) at transplantation	55.6 ± 8.3 (34–70)
Gender	
Male	65 (67)
Female	32 (33)
Underlying liver disease	
HBV	45 (46)
HCV	38 (39)
HBV, HCV coinfection	5 (5)
Non-B, Non-C	9 (10)
MELD score	10.2 ± 3.8 (6–23)
Alpha-fetoprotein level (ng/mL)	15.69 ± 22.82 (3–153)
Pretransplant treatment	
Yes	82 (84)
Hepatectomy	5
Locoregional therapy (RFA, TACE)	57
Hepatectomy + Locoregional therapy	20
No	15 (16)
Tumor downstaging	5 (5)
Tumor recurrence	
Yes	16 (17)
No	81 (83)

Data are presented as numbers (%) or mean ± SD (range)

### Tumor recurrence and risk factors

**Fig. 4:**
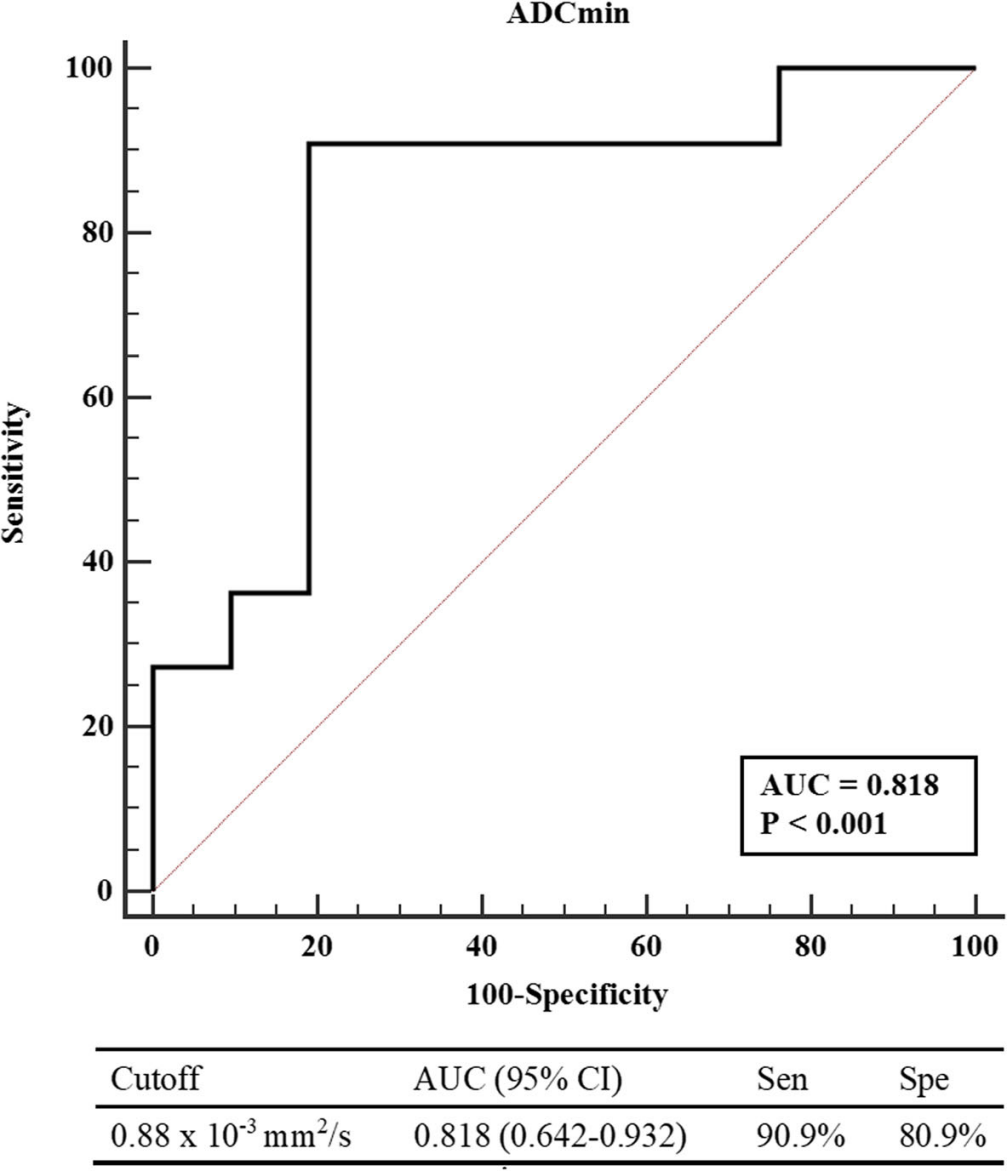
Receiver operating characteristic (ROC) curve of ADC_min_ and the corresponding area under the curve statistics in predicting tumor recurrence

AFP level (15.03 ± 21.60 vs. 19.02 ± 28.85, *p* = 0.526) and largest tumor size (2.83 ± 1.48 vs. 3.15 ± 1.77, *p* = 0.596) were not significantly different between nonrecurrence and recurrence groups (Table 2). Observation of no viable tumor on the imaging before the transplant indicated a lower risk of tumor recurrence (*p* <  0.001). Among the DWI parameters, only the minimum ADC value was a relevant factor (1.07 ± 0.21 vs. 0.78 ± 0.19, *p* = 0.001). The optimal cutoff value for ADC_min_ was ≤0.88 × 10^− 3^ mm^2^/s, and the area under the receiver operating characteristic curve (AUC) was 0.818 (95% confidence interval, 0.642–0.932; Fig. 4). According to the multivariable analysis, as shown in Table 3, ADC_min_ ≤ 0.88 × 10^− 3^ mm^2^/s and the presence of microvascular invasion were independent factors, exhibiting hazard ratios of 13.10 (*p* = 0.022) and 4.49 (*p* = 0.026).

**Table 2:** Disease factors in the nonrecurrence and recurrence groups

Variable	Nonrecurrence (*n* = 81)	Recurrence (*n* = 16)	*P*
Preoperatively available data			
AFP level (ng/mL)	15.03 ± 21.60	19.02 ± 28.85	0.526
Pre-LT treatment			0.721
Yes	68	14	
No	13	2	
Tumors detected on MRI			
Numbers			< 0.001
No viable tumor	60	5	
Within UCSF criteria	21	11	
Largest tumor size (cm)	2.83 ± 1.48	3.15 ± 1.77	0.596
Quantitative indices			
DWI_T/L_	1.31 ± 0.36	1.56 ± 0.41	0.093
ADC_mean_ (× 10^− 3^ mm^2^/s)	1.50 ± 0.27	1.32 ± 0.30	0.111
ADC_min_ (× 10^−3^ mm^2^/s)	1.07 ± 0.21	0.78 ± 0.19	0.001
Postoperatively available data			
Histopathologic features			< 0.001
Hepatocellular carcinoma			
Grade 1–2	79	10	
Grade 3–4	0	3	
HCC + I-CC	1	1	
HCC + cHCC-CC	1	2	
Microvascular invasion			< 0.001
No	72	7	
Yes	9	9	

Data are presented as numbers or mean ± SD

*AFP* Alpha-fetoprotein, *DWI*_*T/L*_ Tumor-to-liver diffusion weighted imaging ratio, *ADC* Apparent diffusion coefficient, *I-CC* Intrahepatic cholangiocarcinoma, *cHCC-CC* Combined hepatocellular cholangiocarcinoma

**Table 3:** Multivariate analysis of prognostic factors associated with tumor recurrence

Variable	HR (95% CI)	*P*
ADC_min_ ≤ 0.88 × 10^− 3^ mm^2^/s	13.10 (1.43–119.54)	0.022
Unfavorable histopathology^a^	4.02 (0.99–16.33)	0.051
Presence of MVI	4.49 (1.19–16.89)	0.026

*ADC* Apparent diffusion coefficient, *MVI* Microvascular invasion

^a^Grade 3–4 HCC, I-CC and cHCC-CC

### Association between DWI parameters and tumor factors

Three of 3 grade 3–4 HCC patients (100%), one of two I-CCC patients (50%), and two of 3 cHCC patients (67%) suffered from tumor recurrence. There was no relationship between the tumor-to-liver DWI ratio (DWI_T/L_) and the risk factors analyzed, including unfavorable histopathology (*p* = 0.359) and microvascular invasion (*p* = 0.942). Also, the mean ADC value did not reach statistical significance (*p* = 0.301 and 0.686). The minimum ADC value was found to be much lower in these aggressive tumors (Table 4).

**Table 4:** Comparison of DWI indices in tumor histopathological findings

	Favorable tumor	Unfavorable tumor^a^	*P*	MVI (−)	MVI (+)	*P*
ADC_min_	1.00 ± 0.26	0.83 ± 0.06	0.004	1.03 ± 0.23	0.83 ± 0.24	0.041
ADC_mean_	1.46 ± 0.31	1.32 ± 0.18	0.301	1.42 ± 0.32	1.47 ± 0.22	0.686
DWI_T/L_	1.37 ± 0.39	1.53 ± 0.38	0.359	1.39 ± 0.40	1.41 ± 0.39	0.942

Data are presented as numbers or mean ± SD

*ADC* Apparent diffusion coefficient, *DWI* Tumor-to-liver diffusion weighted imaging ratio, *MVI* Microvascular invasion

^a^Grade 3–4 HCC, I-CC and cHCC-CC

### ADC_min_ correlation with post-transplant outcome

**Fig. 5:**
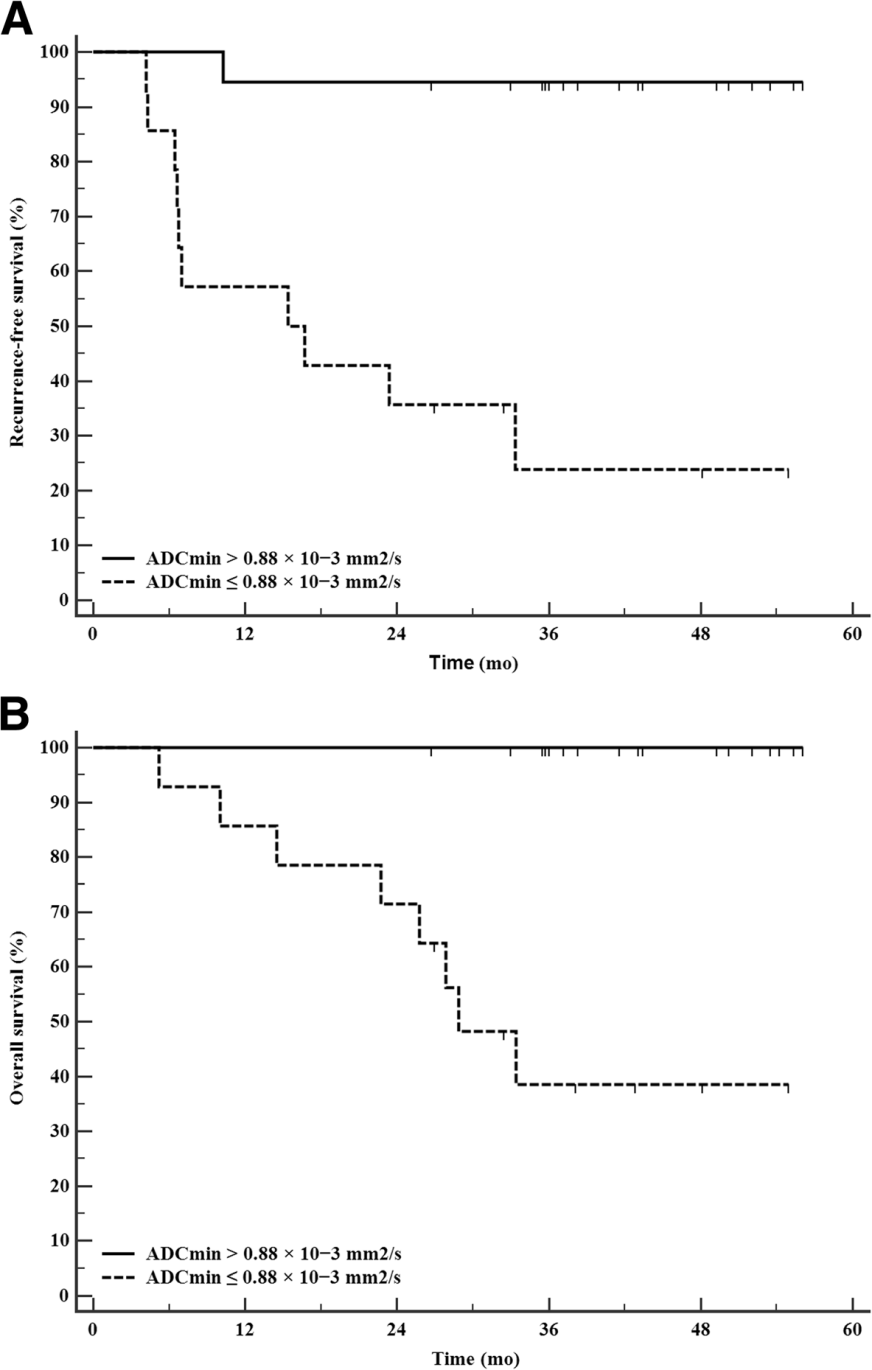
Kaplan–Meier survival analysis according to ADC_min_. **a** One-year and three-year RFS rates were 94.4% vs. 57.1 and 94.4% vs. 23.8%, respectively, for patients with low-ADC and high-ADC tumors. **b** OS rates were 100 and 38.6% for the same two groups

Fourteen patients had low-ADC tumors (ADC_min_ ≤ 0.88 × 10^− 3^ mm^2^/s) showing significantly worse recurrence-free survival (RFS) and overall survival (OS) rates (Fig. 5). The high-ADC group had a 94.4% one-year RFS rate, compared to 57.1% in the low-ADC group. The three-year RFS rates were 94.4 and 23.8%, and the OS rates were 100 and 38.6%.

## Discussion

The results of this study demonstrate the preoperative factors that predict post-transplant outcomes. Our data suggested that the absence of a viable tumor on preoperative imaging was related to lower odds of recurrence (7.6% vs. 34.3%, *p* <  0.001). Similarly to previous studies, neo-adjuvant locoregional therapies were recommended, since these decreased the dropout rates and improved survival [12, 13]. Yao et al. [14] reported an improvement in post-transplant survival for those who received bridging therapy in the subgroup with pT2 and pT3 HCC. The five-year recurrence-free survival was 93.8% versus 80.6%. Furthermore, unsuccessful therapy may indicate the aggressiveness of tumors [15]. The use of pretransplant elevated alpha-fetoprotein level as a predictor of poor prognosis has been increasingly recognized. The upper cutoff values published in several studies vary widely, between 20 ng/mL and 1000 ng/mL [4, 5, 10, 11, 16, 17]. In our practice, we set a cutoff value of 200 ng/mL as an exclusion criterion for transplantation and implemented bridging/downstaging protocols to reduce the tumor burden. Despite this restrictive policy, the recurrence rate remained unsatisfactory, at 17%. Therefore, more detailed information is required for candidate selection.

For patients within UCSF criteria, the minimum ADC values of tumors were significantly lower in the recurrence group (0.78 ± 0.19 vs. 1.07 ± 0.21, *p* = 0.001). Multivariate analysis revealed that an ADC_min_ ≤ 0.88 × 10^− 3^ mm^2^/s was an independent predictor of tumor recurrence (HR = 13.10, *p* = 0.022). DWI combined with ADC mapping has been evaluated as a useful biomarker for tumor featuring and monitoring [18]. ADC_min_ might better reflect the viable solid part of the tumor than ADC_mean_, because increased ADC can be caused by tumor necrosis and arterial reperfusion after TACE. Nakanishi et al. [19] reported that ADC_min_ was significantly lower in patients with early recurrence (0.64 ± 0.24 × 10^− 3^ mm^2^/s, DWI with b values of 50 and 1000 s/mm^2^) after hepatic resection. Lee et al. [20] retrospectively analyzed 114 single HCC patients and found that low ADC_min_ (0.773 × 10^− 3^ mm^2^/s, DWI with b values of 0 and 800 s/mm^2^) was a poor prognostic indicator for RFS. Of particular note is that the cutoff ADC values reported vary between studies. This might be because different study groups use MR scanners of different vendors and specific scan parameters.

Poor tumor differentiation and vascular invasion have been established as major determinants of worse post-liver transplantation (LT) outcomes [21, 22]. In our study, we included two I-CCs and three cHCC-CCs. Vilchez et al. [23], in their analysis of the United Network for Organ Sharing database, found a five-year OS of 40% in cHCC-CC patients, compared to 62% in HCC patients (*p* = 0.002). Another systematic review, by Magistri et al. [24], concluded that LT should be avoided in the management of cHCC-CC. I-CC is typically a contraindication for LT because of inferior survival rates. However, an accurate radiological diagnosis can be difficult, owing to their heterogeneous features. We defined Grade 3–4 HCC, I-CC, and cHCC-CC as unfavorable tumors based on the high recurrence rates (100, 50, and 67%, respectively). Our data demonstrated a good correlation between the minimum ADC value and unfavorable tumors (1.00 ± 0.26 vs. 0.83 ± 0.06, *p* = 0.004). The result confirmed the prior studies, showing an inverse relationship between tumor grade and ADC value. Poorly differentiated HCC shows more restricted diffusion because of its high cellular density, while the ADC values of I-CC tend to be lower due to the presence of abundant desmoplastic stroma in the tumor. But little validation of the range of ADC values specific for I-CC has been documented.

Although macrovascular invasion can be detected with imaging studies, the preoperative prediction of microvascular invasion (MVI) remains elusive. Suh et al. [25] proposed that low ADC values could be applied to the preoperative assessment of MVI, using a cutoff value of 1.11 × 10^− 3^ mm^2^/s. Our data also showed a significantly lower ADC_min_ in tumors with MVI (1.03 ± 0.23 vs. 0.83 ± 0.24, *p* = 0.041). One possible explanation for this finding was the change in microcapillary perfusion. Another possible mechanism was described by Okamura et al. [26], during which they used a higher b-value (1000^s^/mm^2^) and found that restriction of molecular diffusion occurred via an unknown mechanism.

Based on our results, a cutoff value of 0.88 × 10^− 3^ mm^2^/s for ADC_min_ can be recommended for predicting tumor recurrence. The RFS was significantly shorter in the low-ADC group than in the high-ADC group, with a one-year RFS of 57.1 and 94.4% in these two groups, respectively. The overall survival rates were 38.6 and 100%. Low-ADC tumors presented more aggressive biological behavior, which resulted in early recurrence following LT. Apart from tumor morphology evaluated according to the Milan and UCSF criteria, the minimum ADC value of the tumor should be considered as a prognostic factor before deciding on operative management with LT.

## Conclusions

Tumor biology is the most important predictor for post-LT outcome. A low minimum ADC value of viable tumor is associated with higher recurrence after LT and poor survival. Preoperative MR diffusion-weighted imaging therefore provides effective information for the selection of LT candidates.

## Data Availability

The datasets used and/or analyzed during the current study are available from the corresponding author on reasonable request.

## Abbreviations

ADC: Apparent diffusion coefficients
AFP: Alpha-fetoprotein
cHCC-CC: Combined hepatocellular cholangiocarcinoma
DWI: Diffusion-weighted imaging
HBV: Hepatitis B virus
HCC: Hepatocellular carcinoma
HCV: Hepatitis C virus
I-CC: Intrahepatic cholangiocarcinoma
LDLT: Living donor liver transplantation
MELD: Model of end-stage liver disease
MVI: Microvascular invasion
OS: Overall survival
RFA: Radiofrequency ablation
RFS: Recurrence-free survival
TACE: Transarterial chemoembolization
UCSF: University of California San Francisco
